# Changes in Physiological Parameters Induced by Indoor Simulated Driving: Effect of Lower Body Exercise at Mid-Term Break

**DOI:** 10.3390/s90906913

**Published:** 2009-09-01

**Authors:** Wen Chieh Liang, John Yuan, Deh Chuan Sun, Ming Han Lin

**Affiliations:** 1 National Tsing Hua University, Industrial Engineering and Engineering Management 101, Sec. 2, Kuang-Fu Rd., Hsinchu City 300, Taiwan; E-Mail: jyuan916@yahoo.com.tw; 2 Taiwan Scientific Corporation 10F, 88-4, Ming-Chiuan Rd., Shin-Dian City, Taipei 231, Taiwan; E-Mail: sundc@taiwanscientific.com.tw; 3 Ta Hwa Institute of Technology, Automation Engineering No.1, Dahua Rd., Qionglin Shiang Hsinchu County 307, Taiwan; E-Mail: aemhlin@et4.thit.edu.tw

**Keywords:** driving fatigue, heart rate variability (HRV), homeostasis, autonomic nervous system (ANS), simulated driving

## Abstract

The study monitored physiological parameter changes after 120-min of simulated driving. Blood pressures, heart rate (HR), heart rate variability (HRV) and palm temperatures were measured using an ANSWatch^®^ monitor. Subjects were divided into two groups (A & B). Both groups performed 2-hour driving, but group B additionally took a 15-min exercise break. Heart rate, systolic pressure, LF/HF, and palm temperature decreased for group A after driving; for group B only HR and palm temperatures decreased. HRV and parasympathetic indices HF(AU) and HF(NU) increased for group A, while HRV and sympathetic index LF(AU) increased in group B. Group A had higher fatigue scores than group B. ANS activation may overcome some fatigue symptoms, but the recovery is nonetheless incomplete. Exercise break is proven to be an effective remedy, especially if accompanied by the ANS actions. The normalized parasympathetic index HF(NU), the normalized sympathetic index LF(NU), and the sympatho-vagal balance index LF/HF are three most promising parameters that could be further developed to monitor driver fatigue.

## 1. Introduction

Driver fatigue is a vaguely defined term in a physiology sense, but its effect on traffic accidents is well documented. According to a Gallop poll in 2003, driver fatigue was the second most important cause of death (behind driving under the influence of alcohol) in automobile accidents. Nearly a third of survivors that responded to the survey remembered falling asleep while driving. People often have “micro-sleeps” without being aware of them. Numerous statistics and studies have shown that long hours driving resulted in physical tiredness and slowdown in mental judgment. In another report published by Shinar [1], a significant portion of highway accidents was attributed to driver fatigue. The National Transportation Safety Board in the U.S. investigated 286 accidents involving commercial vehicles and discovered that 38% of accidents were caused by the drivers’ drowsiness or distraction. It is believed that a better understanding in driving-associated fatigue on the physiological level may lead to greater awareness of driving risks in general and new development of fatigue preventive devices in particular [1].

Drivers often have a hard time maintaining concentration on a road with repetitious and unexciting scenery. The limited hip and leg space in vehicles also constrain the driver’s lower body from active movement. As a result, the “pumping” action by leg muscle contraction, which assists the venous blood to flow back to the heart, is largely lost [2–3]. It has been our belief that immobilization of the lower body is one of the root causes of driving fatigue. Immobilization not only hinders systemic blood circulation, it also causes body conditions to deviate from homeostasis. We further believe that an effective preventive means or device for driving fatigue and associated traffic accidents must address the problems of blood circulation and homeostasis.

It is well known that when local or systemic circulation is obstructed, the autonomic nervous system (ANS) in the body activates swiftly to compensate. Through its sympathetic and parasympathetic branches, ANS helps the cardiovascular system to maintain proper blood supply under these compromised circumstances [4]. If the activation and execution of ANS is effective, deviations of physiological parameters from the homeostatic states can be avoided. On the other hand, any significant change in vital physiological parameters from baseline may point to an “exhausting” body that is unable to respond to new physiological needs. Eventually, brain hypoxia might occur and sleepiness would soon follow if blood supply to the brain is below basic requirements.

HRV has been used in various studies for the assessment of physiological states. Originated from the sub-conscious cyclic variation in heartbeat period induced by ANS activities, HRV is commonly analyzed in both time and frequency domains to give rise to parameters that are linked to total ANS activity [HRV or SDNN (standard deviation of NN intervals); TP (Total Power)], sympathetic activity [LF(AU) and LF(NU)], parasympathetic (or vagal) activity [HF(AU) and HF(NU)], and sympatho-vagal balance (LF/HF) indexes. Hjortskov *et al.* [5] and Garde *et al.* [6] monitored HRV parameter changes in volunteers before and after a computer task during which various degrees of mental stresses were introduced. The authors reported that the stressors led to changes in HRV [increase in LF(AU), HF(AU), and LF/HF compared to those under resting conditions], and a sustained increase in blood pressures (SYS and DIA). Garde *et al.* [6] reported an increase in heart rate, blood pressure, and LF(NU) and a decrease in TP(AU) and HF(NU) in response to a physically demanding reference computer task. Wahlstrom *et al.* [7] also introduced time and verbal stresses during a mouse-driven computer task to investigate the physiological and psychological changes based upon heart rate, blood pressures (SYS and DIA), and HRV. Increases in both the physiological (HR, BP, LF/HF) and psychological reactions were observed compared to control conditions. These reports suggest that physical and mental stresses may cause the activation of sympathetic nervous system as indicated by increased BP, HR, LF, and LF/HF.

Several authors have investigated the effect of simulated flight on physiological parameters [8–10]. Similar to the indoor driving employed in this study, simulated flight training also involves various degrees of mental stress in a tight-sitting posture. Their general finding is that the complexity of a pilot’s task in operating a flight often caused an increase in HR and BP (SYS and DIA), and a decrease in HRV. Lee *et al.* [9] clearly showed that when the pilots conducted tasks that required high concentration, such as during take-off and landing, their heart rates increased significantly. Among various tasks performed by pilots (take off, climb and cruise, descent and approach, and landing), HRV was seen lowest during approach as it was the most critical period of piloting. It is noted that there are two conditions in simulated flight training sessions that are potentially different from indoor simulated driving studies: they tend to be shorter in duration and more extensive in mental stress.

In the area of indoor simulated driving, Yang *et al.* [11] utilized ECG to monitor the driver’s HRV changes. They discovered four HRV parameters that were significantly changed after driving, namely increased HRV (or SDNN), increased LF(AU), decreased HF(NU), and increased LF/HF. Yang *et al.* [11] also reported that as the degree of fatigue increased (indicated by increasing driving hour), SDNN (equivalent to HRV in this study), LF(NU) and LF/HF all increased while HF(NU) decreased progressively. They believed that the increase in LF/HF was an indication of increase in degree of driving fatigue, as the balance of ANS shifted towards the sympathetic branch. Li *et al.* [12] also based their indoor driving and HRV study on ECG data and found three HRV parameters with significant changes after simulated driving, including increased LF(NU), decreased HF(NU), and increased LF/HF. After three hours of continuous driving, the drivers showed an increase in mental response time, a drop in judgment accuracy, and a lower heart rate. Subjective written questionnaire also showed elevated symptoms of driving fatigue. They proposed using HRV as a quantitative index for driving fatigue. Li *et al.* [13–14] further studied the effect of acupuncture on driving fatigue. Their findings suggested that driving fatigue induced symptoms could be attenuated by acupuncture.

In this study, we monitored multiple physiological parameters, including palm temperatures (left and right), HR, BP (SYS/DIA), and HRV parameters before and after an indoor driving task lasting for 120 minutes. Volunteers were divided into two groups. One group conducted the driving task continuously while the other group had a 15-min exercise break taken at mid-term (60-min from start) of driving. By comparing the physiological changes between the two groups, the effect of exercise break on driving fatigue could be investigated. In addition, a written questionnaire was filled by each participant before and after the driving task to gauge the subjective feeling of driving fatigue. It is our expectation that the results of this series of studies (including Liang *et al.* [15] that was published earlier) could provide useful information for the quantitative definition of driving fatigue based upon physiological parameter changes. Identification of key parameters for monitoring driving fatigue is the next goal for these studies. This is a prerequisite step in the development of driving fatigue preventive devices.

## 2. Materials and Methods

An indoor simulated driving test (instead of a road driving test) was selected in consideration of cost, safety, and control of variables.

### 2.1. Subjects

In order to avoid gender and age influences on HRV [16–17], a total of 40 male subjects within a tight age range (22.6 ± 1.6 years old; see Table 1; all college or graduate school students) were recruited to take the driving test. All subjects were currently healthy and not undergoing any medical treatment. They were instructed to have sufficient sleep in the previous night and not to eat, drink, or exercise one hour prior to the test. All volunteers gave their informed consent before the study. The non-invasive human study was approved by the University Ethics Committee and performed in accordance with the ethical standards outlined in the 1964 Helsinki declaration.

### 2.2. Indoor Simulated Driving

**Figure 1.**
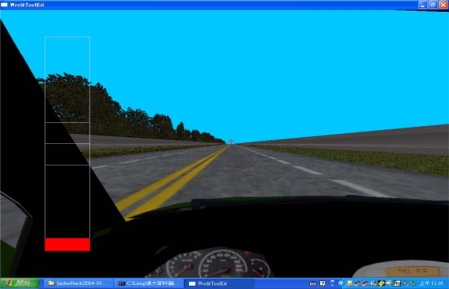
Driving simulator.

The test room was temperature controlled at 22 ± 2 °C. A simulated highway scenery was projected onto a 178 cm × 178 cm white screen using a computer and a projector (Figure 1). The driver’s seat was about 3–4 meters away from the screen. There were randomly arranged trees on the left and walls on the right side of the four-lane, two-way highway. The driver must operate the wheel to keep the vehicle on the designated lanes without hitting trees or walls. A red warning scale appeared on the left side of the windshield which would vertically expand in area if the vehicle location deviated further from the designated lane. Noises or laud sounds would go off if the vehicle got too close to or made contact with road trees or walls. The driving task lasted 120 minutes.

### 2.3. Apparatus

The experimental apparatus was comprised of a notebook computer, a computer projector, a driving wheel, a timer watch, a body weight and fat balance, a high-precision thermometer (±0.1°C), and ANSWatch^®^. Software included SPSS 12.0, highway scenery simulator and an “ANSWatch^®^ Manager Pro” data analyzer.

### 2.4. Procedure

**Figure 2.**
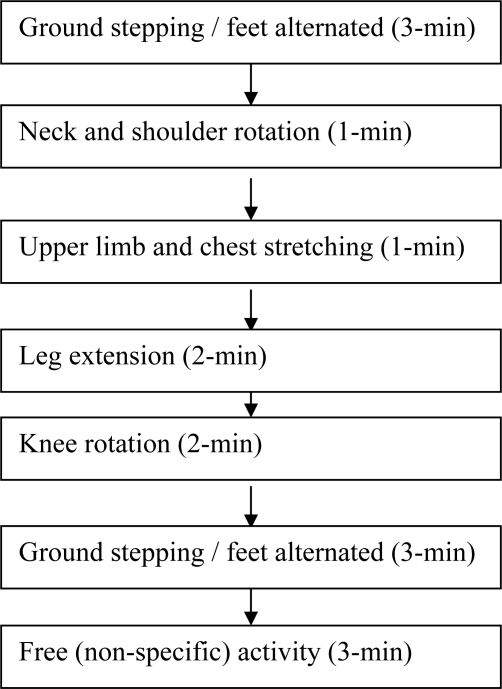
Exercise break procedure.

**Figure 3.**
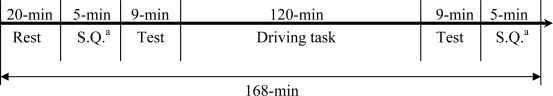
Procedure of group A (w/o exercise). ^a^ : Subjective Questionnaire

**Figure 4.**
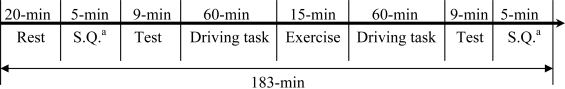
Procedure of group B (with exercise). ^a^ : Subjective Questionnaire

Forty volunteers were randomly divided into two groups (A & B). Both groups performed the 120-min driving task in the afternoon from 1:30∼4:30 PM. Group A conducted the driving test continuously, while group B took a 15-min exercise break at the mid-point of driving (60 min from the start). The exercise included feet-ground alternated stepping, neck and shoulder rotation, upper limb and chest stretching, leg extension, and knee rotation (Figure 2) [2–3]. When they reported to the test room, each volunteer took a 20-min rest first and then underwent thermometer (both palms) and ANSWatch^®^ tests before taking the driving test. The two tests took about seven minutes. Data in ANSWatch^®^ was downloaded to the notebook computer immediately following the test for review and storage. The driving task lasted for 120 minutes for group A and 135 minutes (including 15-min exercise break) for group B, respectively. After driving, each volunteer was tested again for palm temperatures and ANSWatch^®^. In addition, a written questionnaire consisting of 14 questions related to subjective feeling of fatigue was filled by the volunteers shortly before and after driving task [13,18]. The entire testing program is illustrated in Figures 3 and 4. The list of questions is shown in Table 2 while the quantitative scale for the questionnaire is shown in Table 3.

### 2.5. Data Collection

To date, most HRV studies have used Electrocardiography (ECG) data due to its availability in research laboratories. A few studies have based their HRV measurements on finger blood pressure waveforms using an optical sensor [19–21]. The authors reported correlation coefficients of HRV parameters in the range of 0.75 to 0.99 when compared to ECG. In this work, we have been using a new ANSWatch^®^ wrist monitor (Taiwan Scientific Corporation, Taipei, Taiwan; Taiwan Department of Health medical device product registration number 001525) which employs multiple piezo-electrical sensors enclosed in the cuff to directly measure the blood pressure waveforms in the radial artery. According to the company documents submitted to Taiwan Department of Health [22] and published clinical reports [23], the device accuracy on HRV parameters in terms of correlation coefficient is in the range of 0.90 to 1.0, using ECG as the control. This portable device requires neither electrodes nor other disposables, and can conduct tests in sitting or lying (supine) postures. Each ANSWatch^®^ test takes about 7 minutes and outputs eight patient parameters on the LCD screen [HR, SYS, DIA, HRV, LF(NU), HF(NU), sympatho-parasympathetic balance index LF/HF, and number of irregular heartbeats]. Upon data download to a PC and using the accompanied software (ANSWatch^®^ Manager Pro.), more HRV parameters can be calculated [such as total power TP, very low frequency (absolute) VLF(AU), and square root of the mean of the sum of the squares of differences between adjacent peak intervals RMMSD, etc.]

### 2.6. Physiological Parameter Analysis

**Figure 5.**
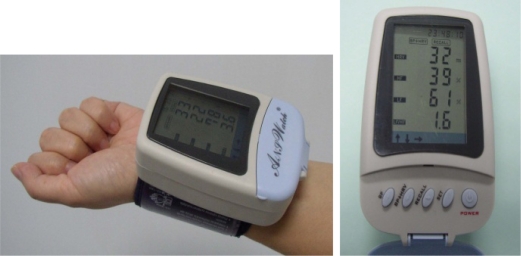
ANSWatch^®^ wrist monitor.

In its standard 7-min test, ANSWatch^®^ (Figure 5) first uses the oscillatory method to obtain heart rate, systolic pressure, and diastolic pressure. It then conducts a standard 5-min HRV test. The piezo-electrical sensors in the cuff pick up blood pressure waveforms produced by the radial artery, with the aid of an air pouch pressure controlled by an air pump. Peak-to-peak intervals are determined followed by time and frequency domain analyses. The HRV analysis follows closely the 1996 international standard (Task Force of the European Society of Cardiology and the North American Society of Pacing and Electrophysiology, 1996) (see Appendix A [24]). Device specifications and detailed analytical procedures were published previously elsewhere [15].

It is noted that irregular heartbeats (those with peak intervals greater than four standard deviations of peak-to-peak intervals during the 5-min test per ANSWatch^®^ definition) are excluded from the raw data prior to HRV analysis (as recommended by the 1996 Standard). The HRV parameters along with associated physiological meanings and units used in the study are listed in Appendix B.

### 2.7. Statistical Analyses

Student’s t tests (two-tailed) were used throughout the entire study to determine the significance of parameter changes before and after the driving task for respective driving groups. Furthermore, one-way MANOVA (multivariate analysis of variance) analyses were used to examine any group difference. The fatigue questionnaire results were analyzed using the same methods.

## 3. Results

### 3.1. Variation in Physiology Parameters before and after Driving (Paired t-test Analysis)

Tables 4 and 5 show the paired t-test results for group A (w/o exercise) and group B (with exercise), respectively.

From Table 4, SYS, HR, LF/HF, left palm temperature T_LP_, and right palm temperature T_RP_ exhibited significant decrease, while HRV, HF(AU), HF(NU), and VLF(AU) showed significant increase after 120-min continuous driving for group A (w/o exercise) (all p < 0.05), while changes in DIA, LF(AU) and LF(NU) did not reach statistical significance.

From Table 5, HR, left palm temperature T_LP_, and right palm temperature T_RP_ exhibited significant decrease, while HRV and LF(AU) showed significant increase after driving task for group B (with exercise) (all p < 0.05), while changes in SYS, DIA, LF(NU), HF(AU), HF(NU), VLF(AU) and LF/HF did not reach statistical significance.

### 3.2. Variation in Physiological Parameters (One-way Multivariate Analysis of Variance, MANOVA) (w/o exercise vs. with exercise)

#### 3.2.1. Before driving

##### 3.2.1.1. Entire group of physiological parameters

This analysis was based upon the entire group of physiological parameters measured before driving in the study (a total of 12, see Table 4 or 5) to examine any statistical difference between group A (w/o exercise) and group B (with exercise). There was no significant difference (Wilks’λ = 0.779, p = 0.792) between group A (w/o exercise) and group B (with exercise) for the 12 physiological parameters obtained before driving.

##### 3.2.1.2. Individual physiological parameters (Independent t-test)

Individual physiological parameters measured before driving were examined by independent t-tests for any group difference between group A (w/o exercise) and group B (with exercise). No significant difference was seen between two groups for any of the 12 physiological parameters measured before driving (data not shown for brevity).

#### 3.2.2. Changes in physiological parameters due to driving

##### 3.2.2.1. Entire group of physiological parameter changes

This analysis was based upon the entire group of physiological parameter changes due to driving measured in the study (a total of 12, see Table 4 or 5) to examine any statistical difference between group A (w/o exercise) and group B (with exercise). The results indicated that significant difference (Wilks’ λ = 0.487, p = 0.03) was observed between group A and group B (p < 0.05) for driving-induced changes of the 12 physiological parameters measured in the study.

##### 3.2.2.2. Individual physiological parameter changes

Examination of individual physiological parameter changes due to driving for any group difference between group A (w/o exercise) and group B (with exercise) was conducted by independent t-tests. Results are shown in Table 6. From Table 6, the results indicated that changes in HF(NU), LF(NU) and LF/HF due to driving showed a statistical difference between group A and group B (p < 0.05). More specifically, HF(NU) was increased due to driving in group A (without exercise) but decreased in group B. LF(NU) and LF/HF were both decreased due to driving in group A but increased in group B. P-value for the change in SYS due to driving was 0.051 between the two groups, very close to being statistically significant. From earlier paired t-tests (Tables 4 and 5), SYS was seen to drop after driving for group A, but maintained for group B.

### 3.3. Subjective Evaluation

It was confirmed by verbal communication that all volunteers had followed the pre-test instructions (having sufficient sleep in the previous night and no eating, drinking, or exercise one hour prior to the test and staying fresh and healthy when reporting to the test laboratory). Before and after the driving task, each volunteer filled the fatigue questionnaire. Results of the written questionnaire for both driving groups, paired t-test, and independent t-test between groups are tabulated in Tables 7–10.

#### 3.3.1. Paired t-test for group A and group B

Paired t-tests of fatigue scores were conducted for both group A (w/o exercise) and group B (with exercise) before and after the driving task. Results are shown in Tables 7 and 8.

##### 3.3.1.1. Group A (w/o exercise)

From Table 7, all fatigue questions except ”Hand and foot trembling” (13 questions out of 14 total questions) showed increased scores after driving for the group A (w/o exercise)(p < 0.05). In addition, average scores and total scores were all significantly higher following driving (p < 0.05). The questionnaire results clearly indicated subjective feeling of driving fatigue felt in these young volunteers after 120-min continuous driving.

##### 3.3.1.2. Group B (with exercise)

From Table 8, nine fatigue questions (out of 14 total questions) showed increased scores after driving for the group B (with exercise)(p < 0.05). The exceptions were “Desire to lie down“, “Shoulder stiffening”, “Waist pain”, “Feeling of vomit”, and “Hand and foot trembling”. In addition, average scores and total scores were all significantly higher following driving (p < 0.05). Similar to the group A, volunteers in the group B also clearly felt driving fatigue after 120-min driving plus a 15-min mid-term exercise break. Judging from number of fatigue score increases (13 for group A; 9 for group B), average score per question (4.12 for group A; 3.44 for group B), and total average score (57.8 for group A; 48.2 for group B), group A volunteers (without exercise break) felt more exhausted than group B (with exercise break). More statistics comparing the two driving groups are discussed below.

#### 3.3.2. Independent t test between two groups before driving

Results of independent t-tests for fatigue scores between the two driving groups before driving indicated that all fatigue questions, average score per question and total average score were all similar between the two groups measured before driving (p > 0.05). Overall, volunteers were not feeling fatigue (almost all scores below 3.0, the threshold for feeling of fatigue, see definition in Table 3) before driving regardless of driving group (data not shown for brevity).

#### 3.3.3. Independent t test between two groups after driving

Results of independent t-tests for fatigue scores between the two driving groups after driving are tabulated in Table 9. From this Table, group A (without exercise break) showed significant higher fatigue scores than group B (with exercise break) in two specific questions (“Loss of concentration” and “Desire to lie down”) (all p < 0.05). In addition, average score per question (4.12 for group A; 3.44 for group B) and total average score (57.8 for group A; 48.2 for group B) all were higher for group A (p < 0.05) after driving. Overall, group A volunteers felt more fatigue than group B after driving.

#### 3.3.4. Independent t-test for driving-induced changes in fatigue scores between driving group

Results of independent t-tests between the two driving groups for the fatigue score changes due to driving are tabulated in Table 10. From Table 10, group A (without exercise break) showed significant higher fatigue score changes due to driving than group B (with exercise break) in four specific questions (“Body tiredness”, “Loss of concentration”, “Desire to lie down”, and “Eye fatigue”) (all p < 0.05). In addition, driving-induced changes in average score per question (2.03 for group A; 1.13 for group B) and total average score (28.45 for group A; 15.90 for group B) were all higher for group A (p < 0.05). Overall, group A volunteers felt more fatigue than group B.

## 4. Discussion

Multiple vital physiological parameters, in addition to a written questionnaire, were monitored in the study to evaluate driving-induced fatigue. Furthermore, an attempt to reduce driving fatigue symptoms was made by the introduction of a 15-min exercise break in group B. Physiological parameter changes and their implications and interactions are discussed below.

Systolic pressure (SYS) was lowered from 116.7 mmHg before driving to 110.2 mmHg after driving for group A (without exercise). In contrast, SYS was almost unchanged (116.7 mmHg before driving and 116.4 mmHg after driving) for group B with the 15-min exercise break. Independent t-test confirmed that SYS was significantly different between the two driving groups after driving (see Section 3.2.2 in Results). Diastolic pressure was little changed in either driving group (Tables 4 and 5). Overall, homeostasis of blood pressure was better maintained in group B with the assistance of the 15-min exercise which was specifically designed to boost lower body and systemic circulation (see Figure 2). The results are in agreement with written questionnaire where group A volunteers felt more exhausted than group B (see Section 3.3.3 in Results). From these results, deviation of systolic pressure from homeostasis could be a candidate for quantitative index of driver fatigue. On the other hand, diastolic pressure change is not a sensitive parameter for monitoring fatigue. Our results differ from those reported by Fumio *et al.* [28] where SYS was found to increase in taxi drivers measured during city road traffic. SYS increase was reported by other authors in various studies where subjects were asked to perform a stress-laden task [5–7,10]. The authors of this study believe that several experimental settings are different between this and those cited studies. Our indoor driving is based upon highway scenery with the objective of simulating multiple-hour driving. The traffic conditions are neither complex nor action-demanding. It normally would not produce high degree of nervousness or anxiety in drivers. On the other hand, poor circulation caused by long-hour lower body immobilization is a characteristic of the experimental setting. In contrast, city road driving tends to be short in duration but requires frequent and spontaneous actions by drivers. Immobilization is not a major concern as drivers often get off the seat to assist passengers. Computer tasks and indoor flight simulations both influence more in mental stress and less in systemic circulation. Consequently, SYS was seen increased in those studies due to increased mental load. One would argue that if those studies were to extend the time of study (into several hours), mental stress would lead to fatigue eventually. One would further expect that SYS would be lowered under these circumstances.

Heart rate (HR) was reduced significantly after driving in both group A (from 70.4 before driving to 65.6 after driving) and group B (from 71.3 before driving to 64.2 after driving). The finding is in agreement with other indoor driving studies [14,18,25] where HR was seen decreased as well. Indoor simulated driving tends to be less stressful compared to actual road driving, due in part to the volunteer’s recognition that the simulation is not real. A stressful condition might have caused an increase in HR. In addition, body movement is limited during simulated driving with the waist, hip, and legs almost stationary. As a combined result, HR is lowered below that under a restful condition. The authors believe that HR decrease is a reflection of poor systematic circulation and decreased cardiac output. Thus, change in HR could be a candidate for quantitative index of driver fatigue.

HRV, an index for total autonomic nervous system (ANS) activity, increased significantly after driving in both group A (from 47.7 ms before driving to 58.6 ms after driving) and group B (from 48.3 ms before driving to 58.7 ms after driving). One can conduct a further analysis by using the mathematic relationship that HRV square is equal to TP (or total power in the frequency domain analysis) which is the summation of HF(AU), LF(AU), and VLF(AU) (all with a unit of ms^2^). From this relationship, the increase in HRV for group A (without exercise) was contributed 78% from very low frequency VLF(AU) (from 1233.6 ms^2^ before driving to 2135.6 ms^2^ after driving), 21% from the parasympathetic activity index HF(AU) (from 506.3 before driving to 757.2 after driving), and 1% from the sympathetic activity index LF(AU) (from 738.3 before driving to 825.5 after driving). By the same analysis for group B (with exercise), HRV increase consisted of 41% contribution from VLF(AU) (from 1550.3 before driving to 1974.0 after driving), 37% from the sympathetic activity index LF(AU) (from 499.8 before driving to 883.5 after driving), and 22% from the parasympathetic activity index HF(AU) (from 638.8 before driving to 875.1 after driving). VLF(AU) was the largest contributor to HRV increase for both group A and group B. Unfortunately its physiological meaning is not defined in the 1996 Standard. For group A, HF(AU) was a significant contributor while LF(AU) was negligible. In contrast, LF(AU)’s contribution (37%) was larger than that of HF(AU) (22%) for group B. The authors attributed the stark difference to the effect of exercise break. It has been observed clinically that during daytime the sympathetic modulation is the dominant mode while during sleep or nighttime the parasympathetic modulation dominates [26–27]. In other words, the sympathetic nervous system is activated when the body activity (mental or physical) is increased. The opposite is true for activation of the parasympathetic nervous system. Furthermore, when the body needs rest due to exhaustion, activation of the parasympathetic branch is called for. In the first publication of this series of studies [15], the same authors compared physiological changes induced by indoor driving conducted in the morning versus in the afternoon. While both driving groups saw a decrease in HR and palm temperatures, and increase in HRV and VLF(AU), three striking differences were observed between the two: (1) LF(AU) and LF(NU) increased while HF(NU) decreased for the morning group; In contrast, LF(NU) decreased while HF(NU) increased for the afternoon group (2) systolic pressure (SYS) was maintained in the morning group but dropped in the afternoon group, and (3) morning group volunteers felt less exhausted than those in the afternoon group as indicated by written questionnaire (all p < 0.05). From the two studies conducted by us, it can be seen that for the driving group that showed higher fatigue scores (the afternoon group in our first study and group A without exercise break in this study), the net effect of ANS was to activate the parasympathetic nervous system, as indicated by an increase in HF(AU) or HF(NU), or a decrease in LF(AU) or LF(NU). Thus, increase in the parasympathetic activity index HF is a good candidate for quantifying driver fatigue. Since LF(NU), HF(NU), and LF/HF are inter-related with well defined mathematic relationship (see Section 2.5), all three are equally good for quantifying driver fatigue. More specifically, the authors propose that an increase in HF(NU), a decrease in LF(NU), or a decrease in LF/HF could be an indication for driver fatigue. The authors prefer using normalized indices of LF and HF (or LF/HF in this sense) instead of absolute counterparts on the ground that they each represent the net effect of ANS activities, rather than the activity of a specific branch. The authors do not believe that change in HRV is a good candidate for driver fatigue correlation. HRV may increase or decrease upon a new physiological need as ANS adjusts the relative contribution of each branch. Thus, the total change in ANS activity does not necessarily represent body fatigue.

Along the same lines, as HR and SYS decreased due to long-hour driving observed in our studies, ANS promptly activated to adjust body conditions for new physiological needs. In the morning versus afternoon study, activation of the sympathetic branch successfully kept SYS from being lowered for the morning group. Volunteers in this group were in their fresh state. Sympathetic stimulation helped to increase cardiac output and contract blood vessels. As a result, SYS was maintained. For the afternoon group in that study, exhaustion must have kicked in to the extent that the body activated the parasympathetic branch to prime for rest. As a result, SYS was lowered. Group A in this study behaved similarly to the afternoon group in the earlier study, as expected. The mid-term exercise break introduced in this study for group B, however, effectively attenuated body exhaustion, as confirmed by questionnaire data analyses (Tables 7–10). With sufficient energy and strength, the body again activated the sympathetic branch (instead of the parasympathetic branch) to meet driving needs. As a result, SYS was maintained for group B. In view of the data shown in Table 5 in this study, physiological behaviors in group B of this study are a lot similar to those recorded for the morning group of our earlier study. In other words, drivers in the afternoon acted like those in the morning with the help of a mid-term exercise break. Our findings here explain well in physiological terms why driving while being tired is highly risky.

The interpretation of our overall findings in this study can be summarized here. As driving fatigue develops, cardio-vascular system is unable to fulfill the basic physiological needs. Hands and feet become cold (T_LP_ and T_RP_ lowered as observed), heart rate slows, and eventually blood pressures go down. Poor circulation causes muscle pain and numbness. Hypoxia in brain induces drowsiness and loss of concentration. Under these circumstances, our body re-assesses the new physiological needs and activates ANS instantly (seen with changes in HRV parameters in the study). If re-assessment finds the body under exhaustion which requires an immediate rest, the parasympathetic nerve branch in ANS is called upon (seen with group A in this study). On the other hand, if re-assessment determines that a boost to cardiovascular system can meet the new physiological needs, the sympathetic nerve branch is activated (group B in this study) (also see [4]). The success of ANS action depends on several factors, including mental alertness, heart strength, and peripheral circulation resistance [5–6,9–10]. Immobilization of the lower body, which increases blood flow resistance significantly, could compromise the sympathetic nerve’s function in boosting systemic circulation [29]. Since the monitoring of physiological parameters was done at the beginning and the ending of driving in the study, it is unclear whether or not the sympathetic nerve branch was activated first in the early face of driving for group A. Noted that at the end of driving for this group, the parasympathetic activity was enhanced. Under these conditions, if the driver continues to drive, the risk of traffic accidents would be extremely high. For group B, the sympathetic nerve was enhanced at the end of driving, which helped to maintain systolic pressure and other vital parameters. But even the ANS action in this group is regarded as a partial success since palm temperatures and heart rate were still below baseline.

The results of this study indicate that driving fatigue can be tracked by vital physiological parameters, including extremity temperatures, heart rate, and blood pressures. Significant deviation of any vital parameter from baseline during or after driving should be viewed as a symptom of fatigue. We further propose that among the HRV parameters recorded, the normalized parasympathetic index HF(NU) (possibly in a positive correlation to fatigue within certain range), the normalized sympathetic index LF(NU) (possibly in a negative correlation to fatigue within certain range), and the sympatho-vagal balance index LF/HF (possibly in a negative correlation to fatigue within certain range) are three most promising parameters that could be further developed to quantify driver fatigue.

## 5. Conclusions

Overall, driving is a demanding task both mentally and physically. ANS activation may overcome some fatigue symptoms, but recovery is nonetheless incomplete. An exercise break is proven to be an effective remedy, especially accompanied by the ANS actions. Among the HRV parameters studied, the normalized parasympathetic index HF(NU), the normalized sympathetic index LF(NU), and the sympatho-vagal balance index LF/HF are three most promising parameters that could be further developed to monitor the driver fatigue.

## Figures and Tables

**Table 1.** Characteristics of subjects.

**Item**	**Average**
Age	22.6 ± 1.6 (years-old)
Body height	1.7 ± 0.05 (m)
Body weight	69.8 ± 9.6 (kg)
BFI ^*^	21.2 ± 4.5 (%)

* Body fat index (BFI) was measured with a commercial Model ULT 2000/ULT 2001 tissue-impedance instrument manufactured by TANITA Corporation (Tokyo, Japan)

**Table 2.** Questionnaire for feeling of driving fatigue.

No	Symptom
1	Body tiredness
2	Loss of concentration
3	Desire to lie down
4	Anxiety
5	Lack of energy
6	Mental response slowdown
7	Headache
8	Shoulder stiffening
9	Waist pain
10	Lower body numbness
11	Eye fatigue
12	Feeling of sleepiness
13	Feeling of vomit
14	Hand and foot trembling

**Table 3.** Quantitative scale for driving fatigue questionnaire.

**Scale**	**Fatigue description**
1	No such feeling
2	Negligible feeling
3	Some feeling
4	Clear feeling
5	Strong feeling
6	Very strong feeling
7	Extremely strong feeling

**Table 4.** Physiological parameters before and after driving for group A.

**Parameters**	**Before driving**	**After driving**	**t-value**	**DF ^a^**	**p-value**
SYS	116.6 ± 7.2	110.2 ± 6.8	−3.48	19	0.002^**^
DIA	74.1 ± 3.0	74.3 ± 2.3	0.44	19	0.664
HR	70.4 ± 8.6	65.6 ± 6.9	−4.00	19	0.001^**^
HRV	47.7 ± 16.9	58.6 ± 17.3	3.53	19	0.002^**^
LF (AU)	738.3 ± 869.5	825.5 ± 590.3	0.49	19	0.625
LF (NU)	59.2 ± 19.0	51.5 ± 17.0	−2.05	19	0.054
HF (AU)	506.3 ± 484.2	757.2 ± 538.2	3.42	19	0.003^**^
HF (NU)	40.6 ± 19.1	48.4 ± 17.0	2.09	19	0.050^*^
VLF (AU)	1233.6 ± 773.1	2135.6 ± 1286.7	3.99	19	0.001^**^
LF/HF	2.0 ± 1.3	1.3 ± 0.9	−2.29	19	0.033^*^
T_LP_	36.5 ± 0.8	35.1 ± 1.6	−5.36	19	0.000^**^
T_RP_	36.5 ± 0.9	35.4 ± 1.7	−3.86	19	0.001^**^

a: DF (degree of freedom)

*: p < 0.05,

**: p < 0.01

**Table 5.** Physiological parameters before and after driving for group B.

**Parameters**	**Before driving**	**After driving**	**t-value**	**DF ^a^**	**p-value**
SYS	116.7 ± 10.1	116.4 ± 9.7	−0.12	19	0.902
DIA	74.4 ± 2.2	74.8 ± 1.9	0.96	19	0.345
HR	71.3 ± 10.6	64.2 ± 8.3	−5.17	19	0.000^**^
HRV	48.3 ± 19.3	58.7 ± 17.2	2.67	19	0.015^*^
LF (AU)	499.8 ± 407.2	883.5 ± 614.8	4.61	19	0.000^**^
LF (NU)	48.7 ± 18.5	53.6 ± 19.0	1.21	19	0.240
HF (AU)	638.8 ± 617.6	875.1 ± 774.5	1.36	19	0.189
HF (NU)	51.2 ± 18.5	46.3 ± 19.0	−1.22	19	0.235
VLF (AU)	1550.3 ± 1612.5	1974.0 ± 1324.1	1.10	19	0.285
LF/HF	1.2 ± 1.0	1.5 ± 1.1	1.30	19	0.209
T_LP_	36.2 ± 0.9	35.4 ± 0.9	−2.76	19	0.012^*^
T_RP_	36.3 ± 0.7	35.6 ± 0.9	−2.60	19	0.017^*^

a: DF (degree of freedom)

*: p < 0.05,

**: p < 0.01

**Table 6.** Comparison between group A (w/o exercise) and group B (with exercise) for individual physiological parameter changes due to driving.

**Parameters**	**Average of change ^c^ due to driving**		**Difference ^b^ between two groups**	**Standard deviation**	**t-value**	**DF ^a^**	**p-value**
	**Group A**	**Group B**					
SYS	−6.40	−0.30	6.10	3.026	2.015	38	0.051
DIA	0.25	0.40	0.15	0.700	0.214	38	0.832
HR	−4.85	−7.10	−2.25	1.831	−1.229	38	0.227
HRV	10.90	10.45	−0.45	4.981	−0.090	38	0.928
HF(AU)	250.95	236.30	14.65	188.283	0.078	38	0.938
HF(NU)	7.80	−4.90	−12.70	5.467	−2.323	38	0.026^*^
LF(AU)	87.15	383.70	296.55	194.207	1.527	38	0.135
LF(NU)	−7.70	4.85	12.55	5.477	2.291	38	0.028^*^
VLF(AU)	902.05	423.75	−478.30	446.174	−1.072	38	0.290
LF/HF	−0.66	0.29	0.95	0.369	2.613	38	0.013^*^
T_LP_	−1.37	−0.81	0.56	0.390	1.434	38	0.160
T_RP_	−1.07	−0.66	0.41	0.377	1.087	38	0.284

a: DF (degree of freedom)

b: Difference was defined as value (group B) – value (group A)

c: Change was defined as value (after driving) – value (before driving)

*: p < 0.05

**Table 7.** Paired t-tests of questionnaire fatigue scores before and after driving for group A.

**Question items**	**Average score**		**Standard deviation**	**t-value**	**DF ^a^**	**p-value**
	**Before driving**	**After driving**				
Body tiredness	2.30	5.15	1.663	7.664	19	0.000^**^
Loss of concentration	2.45	5.20	1.446	8.503	19	0.000^**^
Desire to lie down	2.45	5.20	1.482	8.297	19	0.000^**^
Anxiety	2.20	4.15	1.959	4.451	19	0.000^**^
Lack of energy	2.55	4.50	1.571	5.548	19	0.000^**^
Mental response slowdown	2.20	4.35	1.225	7.844	19	0.000^**^
Headache	1.55	2.55	1.414	3.162	19	0.005^**^
Shoulder stiffening	2.15	4.15	2.077	4.305	19	0.000^**^
Waist pain	2.05	3.35	2.105	2.762	19	0.012^*^
Lower body numbness	1.75	3.80	1.731	5.295	19	0.000^**^
Eye fatigue	2.15	5.40	1.916	7.586	19	0.000^**^
Feelings of sleepiness	2.35	5.50	1.496	9.414	19	0.000^**^
Feelings of nausea	1.40	2.15	1.292	2.595	19	0.018^*^
Hand and foot trembling	1.80	2.35	1.316	1.868	19	0.077

Average score per question	2.09	4.12	1.165	7.800	19	0.000^**^
Total average score	29.35	57.8	16.311	7.800	19	0.000^**^

a: DF (degree of freedom)

*: p < 0.05,

**: p < 0.01

**Table 8.** Paired t-tests of questionnaire fatigue scores before and after driving for group B.

**Question items**	**Average score**		**Standard deviation**	**t-value**	**DF ^a^**	**p-value**
	**Before driving**	**After driving**				
Body tiredness	2.75	4.25	2.013	3.332	19	0.004^**^
Loss of concentration	2.80	4.05	1.888	2.960	19	0.008^**^
Desire to lie down	3.00	4.05	2.327	2.017	19	0.058
Anxiety	2.15	3.20	1.700	2.761	19	0.012^*^
Lack of energy	2.75	4.00	2.268	2.465	19	0.023^*^
Mental response slowdown	2.70	3.85	1.899	2.708	19	0.014^*^
Headache	1.45	2.50	2.064	2.275	19	0.035^*^
Shoulder stiffening	2.55	3.45	2.845	1.415	19	0.173
Waist pain	1.90	2.45	2.305	1.067	19	0.299
Lower body numbness	2.05	3.05	1.806	2.476	19	0.023^*^
Eye fatigue	2.85	4.90	1.761	5.205	19	0.000^**^
Feelings of sleepiness	2.60	4.95	2.345	4.480	19	0.000^**^
Feeling of nausea	1.25	1.70	1.669	1.206	19	0.243
Hand and foot trembling	1.50	1.80	1.128	1.189	19	0.249

Average score per question	2.30	3.44	1.397	3.633	19	0.002^**^
Total average score	32.30	48.20	19.571	3.633	19	0.002^**^

a: DF (degree of freedom)

*: p < 0.05,

**: p < 0.01

**Table 9.** Comparison between group A and group B for fatigue scores after driving.

**Question items**	**Average score after driving**		**Difference ^a^ between group**	**t-value**	**DF ^b^**	**p-value**
	**Group A**	**Group B**				
Body tiredness	5.15	4.25	−0.90	2.009	38	0.052
Loss of concentration	5.20	4.05	−1.15	2.992	38	0.005^**^
Desire to lie down	5.20	4.05	−1.15	2.136	38	0.039^*^
Anxiety	4.15	3.20	−0.95	1.818	38	0.077
Lack of energy	4.50	4.00	−0.50	1.070	38	0.291
Mental response slowdown	4.35	3.85	−0.50	1.082	38	0.286
Headache	2.55	2.50	−0.05	0.091	38	0.928
Shoulder stiffening	4.15	3.45	−0.70	1.147	38	0.259
Waist pain	3.35	2.45	−0.90	1.530	38	0.134
Lower body numbness	3.80	3.05	−0.75	1.575	38	0.124
Eye fatigue	5.40	4.90	−0.50	1.060	38	0.296
Feelings of sleepiness	5.50	4.95	−0.55	1.214	38	0.232
Feelings of nausea	2.15	1.70	−0.45	1.001	38	0.323
Hand and foot trembling	2.35	1.80	−0.55	1.436	38	0.159

Average score per question	4.12	3.44	−0.68	2.149	38	0.038^*^
Total average score	57.8	48.20	−9.60	2.149	38	0.038^*^

a: Difference was defined as value (group B) – value (group A)

b: DF (degree of freedom)

*: p < 0.05,

**: p < 0.01

**Table 10.** Comparison between driving groups for fatigue score changes due to driving.

**Question items**	**Average of change^a^ due to driving**		**Difference^b^ between group**	**t-value**	**DF^c^**	**p-value**	
	**Group A**	**Group B**					
Body tiredness	2.85	1.50	1.35	2.312	38	0.026^*^
Loss of concentration	2.75	1.25	1.50	2.820	38	0.008^**^
Desire to lie down	2.75	1.05	1.70	2.755	38	0.009^**^
Anxiety	1.95	1.05	0.90	1.551	38	0.129
Lack of energy	1.95	1.25	0.70	1.134	38	0.264
Mental response slowdown	2.15	1.15	1.00	1.978	38	0.055
Headache	1.00	1.05	−0.05	−0.089	38	0.929
Shoulder stiffening	2.00	0.90	1.10	1.396	38	0.171
Waist pain	1.30	0.55	0.75	1.074	38	0.289
Lower body numbness	2.05	1.00	1.05	1.877	38	0.068
Eye fatigue	3.25	2.05	1.20	2.062	38	0.046^*^
Feelings of sleepiness	3.15	2.35	0.80	1.286	38	0.206
Feelings of nausea	0.75	0.45	0.30	0.635	38	0.529
Hand and foot trembling	0.55	0.30	0.25	0.645	38	0.523

Average score per question	2.03	1.13	0.89	2.203	38	0.034^**^
Total average score	28.45	15.90	12.55	2.203	38	0.034^**^

a: Change was defined as value (after driving) – value (before driving)

b: Difference was defined as value (group A) – value (group B)

c: DF (degree of freedom)

*: p < 0.05,

**: p < 0.01

## Appendix A

The HRV analysis follows closely the 1996 international standard [24] and consists mainly of nine steps:

1. The original data was fed through a low pass FIR filter at 0 to 14 Hz.
2. The original data was fed through a low pass FIR filter at 0 to 14 Hz.
3. The primary peak in each cycle was determined.
4. Peak-to-peak intervals were calculated.
5. Time-domain HRV parameters (mean period or heart rate; variance and standard deviation of peak-to-peak intervals) were calculated. Peak intervals greater than 4*standard deviation were removed and not replaced.
6. Peak-to-peak intervals were re-sampled to 1024 points with interpolation and hamming window adjustment.
7. Fast Fourier Transform (FFT) was performed with hamming window adjustment.
8. Integrations of power spectral density between 0.0001 and 0.04 Hz for the very low frequency component (VLF), between 0.04 and 0.15 Hz for the low frequency component (LF), and between 0.15 and 0.4 Hz for the high frequency component (HF) respectively were conducted.
9. Frequency-domain HRV parameters [VLF (AU), LF (AU), HF (AU), LF (NU), and HF (NU)] were calculated.

## Appendix B

1. HR: Heart rate (beat/min)
2. HRV: Total ANS activity index (ms); equal to standard deviation of adjacent peak-to peak intervals SDNN defined in 1996 standard
3. VLF (AU): Very low frequency (0.0001∼0.04 Hz) (ms^2^)
4. LF (AU): Low frequency (0.04∼0.15 Hz) (ms^2^); sympathetic nervous activity
5. HF (AU): High frequency (0.15∼0.4 Hz) (ms^2^); parasympathetic nervous activity
6. LF (NU): [LF/(TP-VLF)]*100; contribution of sympathetic nervous activity
7. HF (NU): [HF/(TP-VLF)]*100; contribution of parasympathetic nervous activity
8. LF/HF : Ratio of LF to HF; sympatho-vagal balance

Although the physiological meaning for VLF (AU) is not defined in the 1996 Standard [24], it has been reported and cited by several other authors. We decided to include it in the report for completeness.
